# Targeting telomeres in brain vasculature does not impede initiation and progression of glioblastoma

**DOI:** 10.1371/journal.pone.0355168

**Published:** 2026-08-12

**Authors:** Amparo Sánchez-Hernández, Sonia Burgaz, Jessica Louzame-Ruano, Óscar Laguía, Giuseppe Bosso, Rosa Serrano, Ana Cayuela López, Juana María Flores, Paula Martínez, Maria A. Blasco

**Affiliations:** 1 Telomeres and Telomerase Group, Molecular Oncology Program, Spanish National Research Cancer Centre (CNIO), Madrid, Spain; 2 The Confocal Microscopy Unit, Spanish National Research Cancer Centre (CNIO), Madrid, Spain; 3 Animal Surgery and Medicine Department, Faculty of Veterinary Science, Complutense University of Madrid, Madrid, Spain; University of Newcastle, UNITED KINGDOM OF GREAT BRITAIN AND NORTHERN IRELAND

## Abstract

The tumor microenvironment is proposed to have an essential role in the growth and therapeutic response of glioblastoma. Vessel formation or angiogenesis has been an important target of different therapeutic strategies. In the past, we described that targeting telomere protection through abrogation or inhibition of the TRF1 shelterin telomere protein in a GBM model is sufficient to delay tumor growth and progression. Here, we set out to address whether *Trf1* deletion only in endothelial cells has an impact on GBM growth and progression. Although we saw a tendency to a decrease in CD31-positive cells in GBM tumors where *Trf1* was deleted, which was concomitant with a tendency to increased global DNA damage and increased telomere induced DNA damage foci in endothelial cells, as expected from telomere unprotection, this was not sufficient to delay tumor growth and progression. These findings suggest that TRF1-dependent telomere protection in endothelial cells is not a major limiting factor for PDGF-driven GBM at tumor initiation and progression.

## Introduction

Malignant gliomas represent the majority of primary central nervous system (CNS) neoplasms. The most frequent and aggressive glioma is glioblastoma (GBM) [1]. In recent years, despite the advances in the molecular characterization of glioblastoma and improvements in therapeutic approaches, including the Stupp protocol [2] and Tumor Treating Fields (TTFields) [3], patients prognosis remains poor, with a median survival of approximately 14–16 months [4].

GBM is known for its high proliferative and infiltrative nature [5]. GBMs are also highly heterogeneous tumors [6]. Cells within the tumor present different expression profiles and may have different responses to radio- and chemotherapy [7]. Among these cell populations, endothelial cells are of particular importance as they contribute to the high vascularization of these tumors, and support the tumor microenvironment by connecting tumor cells with infiltrating immune cells, glioma stem cells, and the extracellular matrix, which drives tumor progression [8]. Many therapeutic strategies have been developed to treat tumor angiogenesis in GBM. In recent years, the exploration of immunotherapeutic strategies has arisen as a potential strategy to enhance GBM treatment outcomes. While these approaches show promise, the immunosuppressive tumor microenvironment and high heterogeneity of GBM remain significant challenges [9–11]. Despite significant progress in understanding the molecular aspects of glioblastoma, new therapeutic approaches are needed.

Telomeres are protective structures at the end of chromosomes essential for chromosome stability [12]. Mammalian telomeres are formed by tandem repeats of the TTAGGG sequence bound by the so-called shelterin complex, formed by TRF1, TRF2, POT1, TPP1, TIN2, and RAP1 [12,13]. With each cell division, telomeres shorten due to the incomplete replication of chromosome ends, a phenomenon known as the “end-replication” problem [14]. This telomere shortening can be compensated through the *de novo* addition of telomeric repeats by telomerase, a reverse transcriptase composed of a catalytic subunit (Telomerase Reverse Transcriptase or TERT) and an RNA component (Telomerase RNA component or Terc), used as a template for the synthesis of TTAGGG repeats [15]. In normal adult cells, however, telomerase is not usually expressed and telomeres progressively shorten associated with tissue repair and, thus, to organismal aging [14,16]. Telomere maintenance above a minimum length is essential to sustain the indefinite proliferation potential of cancer cells, thus telomeres are considered as potential anti-cancer targets [17–19]. More than 90% of human tumors aberrantly express telomerase [20], while the remaining telomerase-negative tumors activate an alternative mechanism to elongate telomeres based on recombination between telomeric sequences, known as ALT [21]. The promoter of the *TERT* gene is mutated in 58–84% of human primary GBMs [22,23], while pediatric GBMs frequently display an ALT phenotype associated with *ATRX* mutations [24]. In addition to telomerase, mutations in shelterin proteins have been found in familial glioblastoma [25]. In particular, dominant negative mutations in POT1 have been found in different tumor types, including familial glioblastoma cases, again highlighting the importance of telomeres in GBM [25–28]. These facts highlight the importance of telomere maintenance in glioblastoma and pinpoint telomeres as promising targets.

In our group, we have shown that directly targeting shelterin proteins could be effective in targeting telomeres. In particular, we have shown that TRF1 abrogation blocks glioblastoma formation as well as the growth of aggressive and rapidly growing lung tumors in *Trp53*-deficient *Kras*G12V-mice, in a manner that is independent of telomere length, thus further supporting that TRF1 could be a good anti-cancer target for aggressive tumors [19,29–31]. Additionally, targeting TRF2 in glioma stem cells increases their sensitivity to temozolomide *in vitro* and reduces tumorigenesis in xenograft mouse models [32].

Increasing evidence indicates that the tumor microenvironment plays a crucial role in this resistance, with vascular endothelial cells being major contributors. In this regard, therapeutic strategies against GBM have been tried in the past with a limited success. The molecular understanding of the role of vasculature in GBM, thus, remains debated. Notably, hTERT expression has been detected in endothelial cells during the early stages of astrocytic tumor progression, preceding endothelial proliferation and potentially marking the onset of dedifferentiation [33]. In line with these observations, inhibition of telomerase in endothelial cells of GBM xenografts impair angiogenesis, highlighting the critical role of telomerase in astrocytic tumor vascular remodeling [34].

Here, we set out to address whether TRF1 inhibition specifically in endothelial cells blocks GBM initiation and growth in *in vivo* mouse models.

## Results

### *Trf1* genetic deletion in endothelial cells in a GBM model

**Fig 1:**
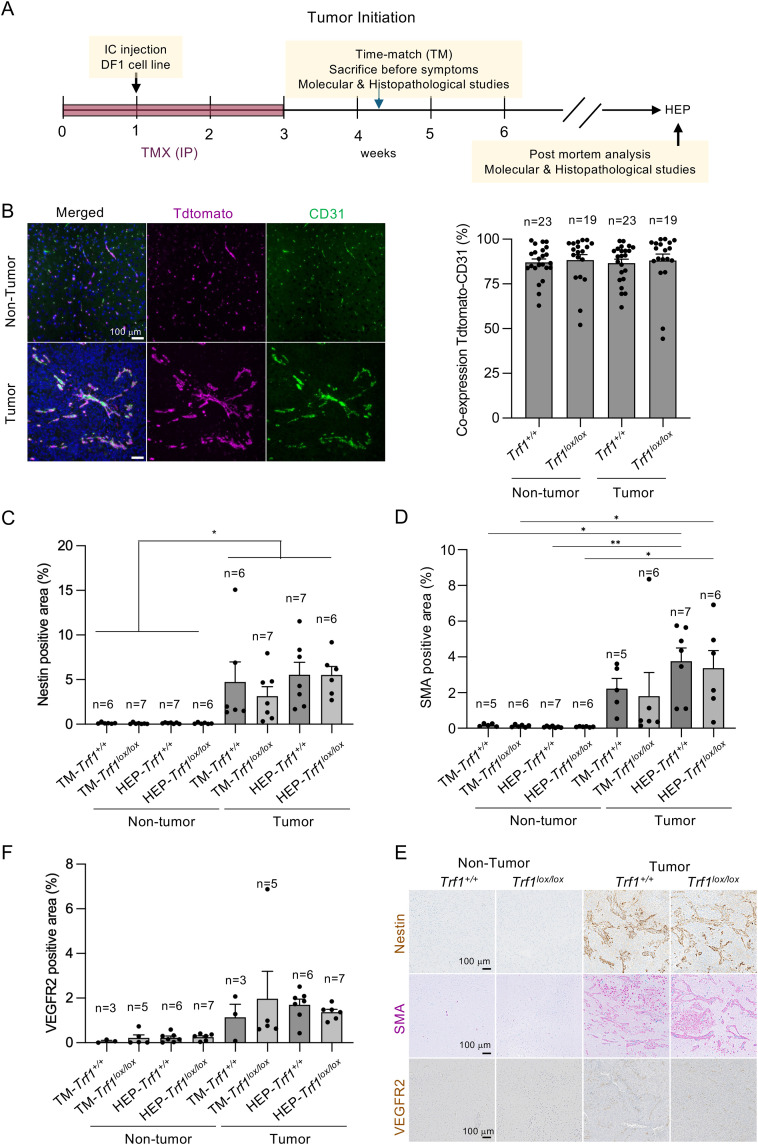
Endothelial-specific deletion of *Trf1* does not affect glioblastoma initiation. **(A)** Schematic representation of the experimental lay-out for the glioblastoma initiation model. *Trf1*^*lox/lox*^ *Nestin-Tva Ink4a/Arf*^*-/-*^ *VE-Cadherin-CreERT2 R26-CAG-tdTomato* mice were treated with tamoxifen (TMX) during three weeks to induce *Trf1* deletion specifically in endothelial cells. After one week on TMX, RCAS–PDGFB–producing DF-1 cells were intracranially injected to induce glioblastoma. Brains were collected at a time-matched (TM) pre-symptomatic stage at day 30 post-induction or at the humane endpoint (HEP) for molecular and histopathological analyses. **(B)** Representative immunofluorescence images of non-tumoral and tumoral brain areas showing tdTomato (red) and the endothelial marker CD31 (green). DAPI was used for nuclear staining. Merged images confirm Cre-mediated recombination specifically in endothelial cells. Quantification of tdTomato/CD31 co-expression is shown on the right. **(C–F)** Quantification of the percentage of Nestin-positive cells (C), SMA-positive area (D), and VEGFR2-positive cells (F) in non-tumoral and tumoral regions from *Trf1*^*+/+*^ and *Trf1*^*lox/lox*^ mice at TM and HEP. **(E)** Representative images of Nestin, SMA, and VEGFR2 staining in brain samples at HEP. Data are shown as mean ± SEM; individual dots represent independent mice. Statistical significance was addressed by one-way ANOVA with Tukey’s multiple comparison test, *p < 0.05; **p < 0.01; ***p < 0.001.

To decipher the effect of *Trf1* deletion in endothelial cells in the brain, which are key players in brain tumor microenvironment and tumor growth [35], we set out to specifically abrogate TRF1 in endothelial cells in the context of a glioblastoma mouse model previously used by us to demonstrate a role for TRF1 in tumor growth [19]. To this end, we crossed the previously generated by us conditional *Trf1*^*lox/lox*^ *Nestin-Tva Ink4Arf*^*-/-*^ glioblastoma mouse model with the *VE-Cadh-CRE.ERT2; R26-CAG-tdTomato* mice, to specifically delete *Trf1* in endothelial cells (Fig 1A) [19]. The tdTomato reporter allows to visualize the TRF1-depleted cells.

By staining with antibodies against the tdTomato reporter and the blood vessel marker CD31, we confirmed Cre expression specifically in endothelial cells as indicated by the colocalization of the tdTomato reporter with the endothelial marker CD31 (Fig 1B).

### Telomere dysfunction owing to TRF1 abrogation in endothelial cells does not impair glioblastoma formation

Next, we induced glioblastoma formation in both *Trf1* deleted or *Trf1* wild-type mice in endothelial cells. To this end, we injected intracranially into the subventricular zone (SVZ) of adult mice (4.5–6.5 weeks-old) the RCAS-PDGFB DF-1 producing cells, specifically targeting Nestin-positive glial progenitors. This results in overexpression of PDGFB in glial progenitors with an *Ink4Arf*^*-/-*^ background, leading to the formation of mesenchymal-subtype GBM from 4 weeks post-intracranial injection (Fig 1A).

In order to delete *Trf1* in endothelial cells, *Trf1*^*lox/lox*^*; Nestin-Tva*; *Ink4Arf*^*-/-*^; *VE-Cadh-CRE.ERT2; R26-CAG-tdTomato* mice were treated with tamoxifen for 4 weeks, starting one week prior to intracranial injection of the RCAS-PDGFB DF-1 producing cells (Fig 1A). We analyzed the percentage of cells positive for Nestin, Smooth Muscle Actin (SMA) and of Vascular Endothelial Growth Factor Receptor 2 (VEGFR2), markers of neural stem cells, activated myofibroblasts and of angiogenesis, respectively, in postmortem brains (Fig 1C-F). The results clearly show an increase in these markers in tumors as compared to healthy brain tissue, demonstrating the glioblastoma nature of the tumors. However, although there was a tendency to lower nestin-positive cells and lower SMA-positive cells in the normal tissue, no differences were observed between wild-type and *Trf1*-deleted tissues (Fig 1C-F).

**Fig 2:**
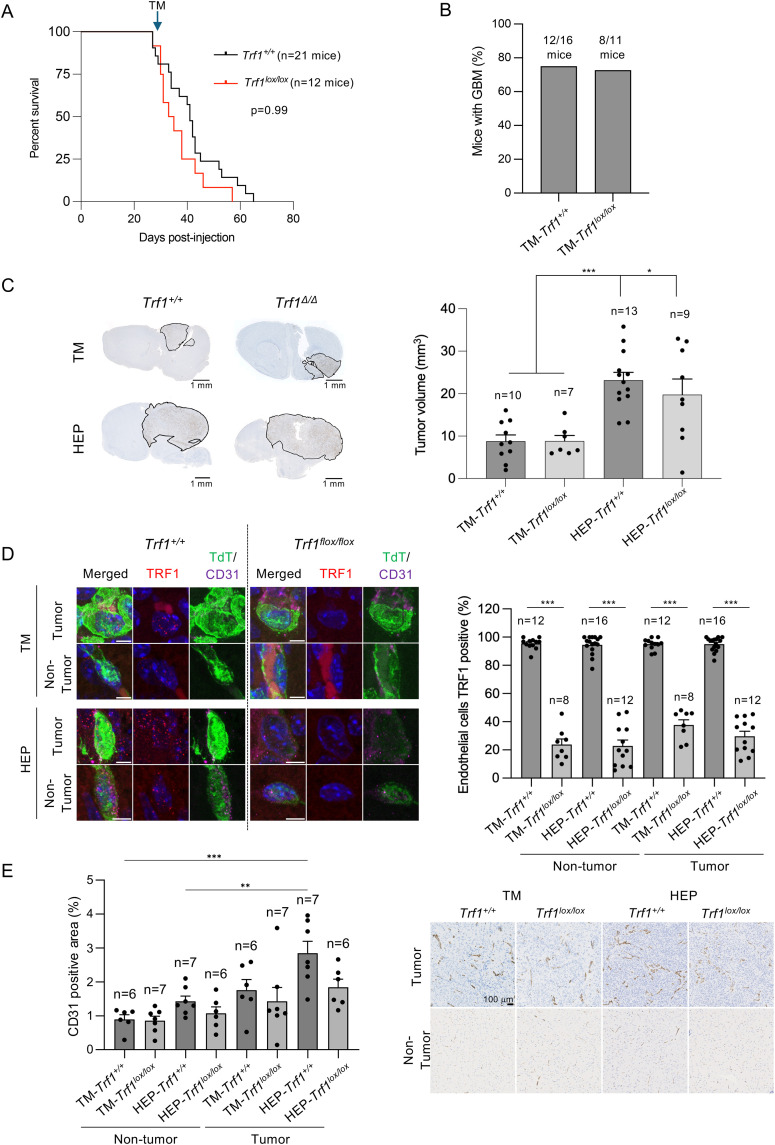
Endothelial *Trf1* deletion does not alter glioblastoma incidence, survival, or tumor burden. **(A)** Kaplan–Meier survival curves of *Trf1*^*+/+*^ and *Trf1*^*lox/lox*^ mice following glioblastoma induction in the initiation model. No significant differences were observed (log-rank test). **(B)** Percentage of mice developing glioblastoma at the time-matched time-point in both genotypes. **(C)** Representative KI67-stained brain sections from *Trf1*^*+/+*^ and *Trf1*^*lox/lox*^ mice at TM and HEP. Tumor areas are marked with dashed lines. Quantification of tumor area is shown on the right. **(D)** Representative confocal images showing TRF1 (red), tdTomato (green), and CD31 (purple) staining in non-tumoral and tumoral regions, confirming efficient and endothelial-specific *Trf1* deletion. Quantification of the percentage of TRF1-positive endothelial cells in non-tumoral and tumoral tissues is shown to the right. **(E)** Representative CD31 immunohistochemistry-stained brain sections from *Trf1*^*+/+*^ and *Trf1*^*lox/lox*^ mice at TM and HEP. Quantification of CD31-positive area in non-tumoral and tumoral tissues is shown to the left. Data are shown as mean ± SEM; individual dots represent independent mice. Statistical significance was addressed by one-way ANOVA with Tukey’s multiple comparison test, *p < 0.05; **p < 0.01; ***p < 0.001.

Of relevance, we did not see differences in mouse survival between both cohorts upon glioblastoma induction (Fig 2A). In agreement with this finding, we observed a similar percentage of mice presenting glioblastoma in both mouse cohorts both at 4 weeks upon induction as well as at the human end point (Fig 2B). Furthermore, the tumor size was also similar in mice with *Trf1* deleted *or Trf1* wild-type in endothelial cells both at 4 weeks upon induction and at the human end-point (Fig 2C).

We confirmed TRF1 deletion specifically in endothelial cells both in normal tissue and tumors at 4 weeks upon GBM induction and at the humane endpoint by staining with TRF1, tdTomato, and CD31 antibodies (Fig 2D).

At the cellular level, we confirmed increased vessel formation in tumor areas compared to non-tumoral areas, as indicated by CD31-positive staining in both wild-type and *Trf1* deleted tumors compared with normal tissue (Fig 2E). Of notice, we observed a tendency to lower vessel formation as determined by CD31-possitive cells in the *Trf1* deleted mice both in the non-tumoral and in the tumoral tissue at the human end point (Fig 2E).

### Telomere dysfunction owing to TRF1 abrogation in endothelial cells induces increased DNA damage in the absence of changes in telomere length

**Fig 3:**
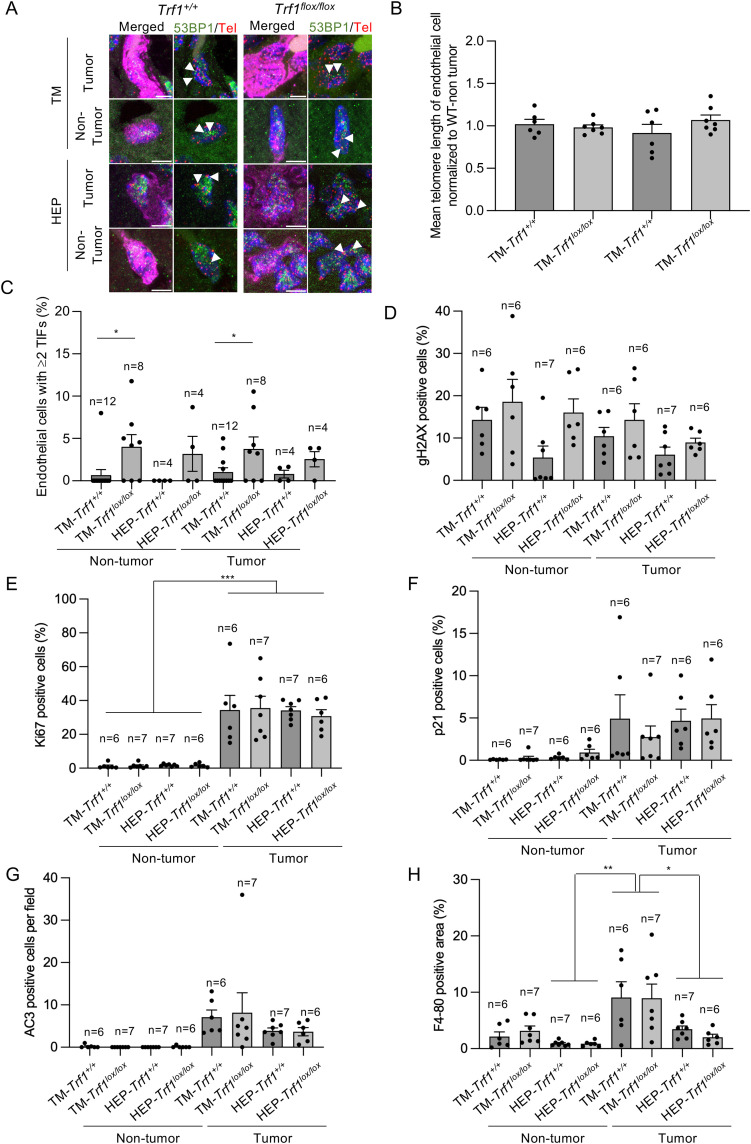
Endothelial *Trf1* deletion induces telomere-specific DNA damage without affecting telomere length or tumor cell proliferation. **(A)** Representative immuno–telomere Q-FISH images showing 53BP1 (green), telomeres (red), and tdTomato (purple) in endothelial cells from *Trf1*^*+/+*^ and *Trf1*^*lox/lox*^ mice at TM and HEP. Arrowheads indicate telomere dysfunction–induced foci (TIFs). **(B)** Quantification of mean telomere length in endothelial cells normalized to *Trf1*^*+/+*^ non-tumoral tissue at TM. **(C)** Percentage of endothelial cells presenting more than two TIFs at TM and HEP. **(D–H)** Quantification of γH2AX-positive cells (D), Ki67-positive cells (E), p21-positive cells (F), cleaved caspase-3–positive cells (G), and F4/80-positive cells (H) in non-tumoral and tumoral regions. Data are shown as mean ± SEM; individual dots represent independent mice. Statistical significance was addressed by one-way ANOVA with Tukey’s multiple comparison test. For TIFs analysis, unpaired non-parametric t-test was performed *p < 0.05; **p < 0.01; ***p < 0.001.

At the molecular level, we observed that TRF1 abrogation in endothelial cells did not affect telomere length in the tumors and in the normal brain tissue (Fig 3A), in agreement with previous findings from our group and others showing that TRF1 abrogation does not result in detectable telomere length changes in different mouse tissues [19,29].

Next, we sought to assess telomeric DNA damage, as indicated by telomere dysfunction-induced foci (TIFs). TIFs were detected by colocalization between telomeres and the DNA damage marker 53BP1 protein. As expected, we observed a tendency towards increased DNA damage specifically located at telomeres, as indicated by increased TIFs in mice with *Trf1* deleted compared to *Trf1* wild-type in endothelial cells (Fig 3B,C). We also saw a tendency to increased global DNA damage as indicated by the percentage of γ-H2AX-positive cells. There were no changes in cell proliferation as indicated by a similar percentage of Ki67-positive cells, in the cell cycle inhibitor p21, in apoptosis as indicated by Caspase 3 staining, nor in the inflammation marked F4-80 in both cohorts in normal and tumoral tissue (Fig 3D-H).

Next, we determined γH2AX and the cell cycle inhibitor p21 specifically in CD31-positive endothelial cells within the tumors. In this case, we did not see differences in the amount of DNA damage and cell cycle arrest as determined by γH2AX-positive and p21 positive cells, respectively, in *Trf1* deleted mice compared to *Trf1* wild-type mice (S1 Fig).

### Telomere dysfunction owing to TRF1 abrogation in endothelial cells in a glioblastoma progression model

**Fig 4:**
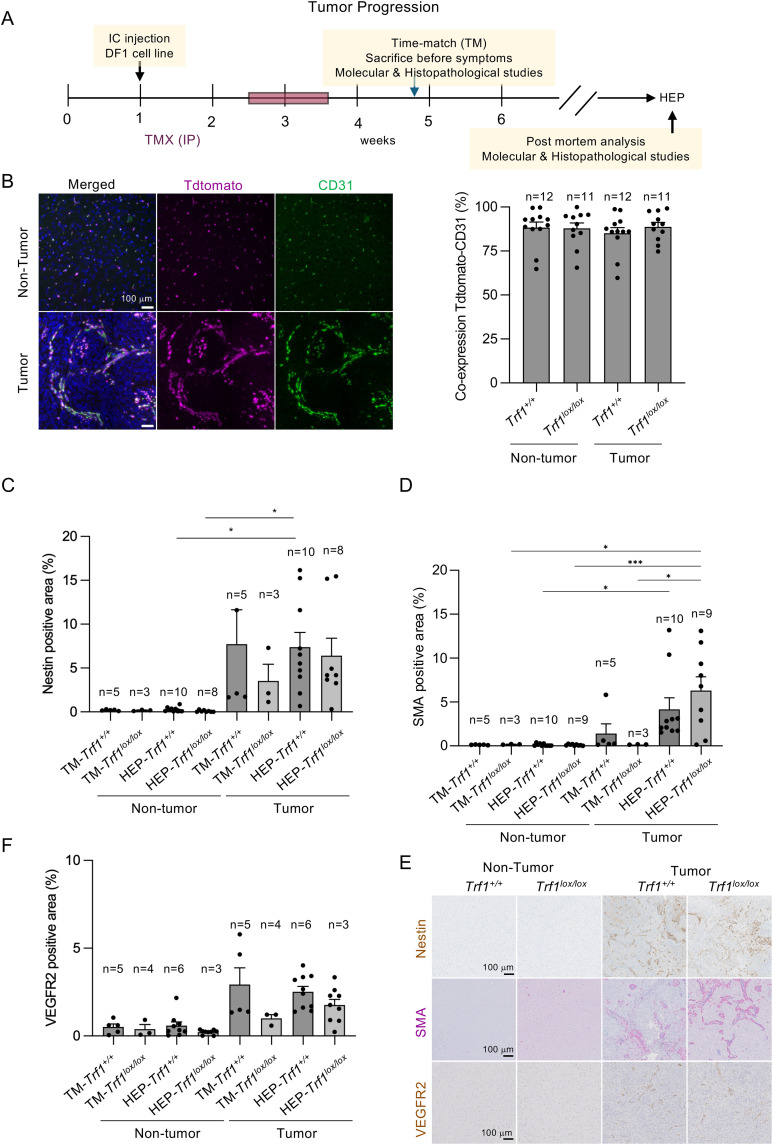
Experimental strategy for endothelial Trf1 deletion during glioblastoma progression. **(A)** Schematic representation of the experimental lay-out for the glioblastoma progression model. Tumors were first induced by intracranial injection of RCAS–PDGFB–producing DF-1 cells. After 2.5 weeks, tamoxifen was administered during one week to delete *Trf1* in endothelial cells. **(B)** Representative immunofluorescence images of tdTomato (red) and CD31 (green) in non-tumoral and tumoral areas, confirming endothelial-specific Cre recombination. Quantification of tdTomato/CD31 co-expression is shown on the right. **(C–F)** Quantification of Nestin-positive cells (C), SMA-positive area (D), and VEGFR2-positive cells (F) in non-tumoral and tumoral tissues from *Trf1*^*+/+*^ and *Trf1*^*lox/lox*^ mice at TM and HEP. **(E)** Representative images of Nestin, SMA and VEGFR2 staining in brain samples at HEP. Data are shown as mean ± SEM; individual dots represent independent mice. Statistical significance was addressed by one-way ANOVA with Tukey’s multiple comparison test, *p < 0.05; **p < 0.01; ***p < 0.001.

Next, to determine the role of endothelial cells in glioblastoma progression, we induced glioblastoma formation first and then deleted *Trf1* in endothelial cells (Fig 4A). To this end, we used the same mice as described above. Tumors were first induced by intracranially introducing into the subventricular zone (SVZ) the RCAS-PDGFB DF-1 producing cells. Then, 2.5 weeks after intracranial injection, mice were intraperitoneally injected with tamoxifen 5 times during a week to delete *Trf1* specifically in the endothelial cells. At this time point, tumors have already started to form, as previously demonstrated by us (Bejarano et al., 2017) (Fig 4A).

By staining with antibodies against the tdTomato reporter and the blood vessel marker CD31, we confirmed *Cre* expression specifically in endothelial cells as indicated by the colocalization of the tdTomato reporter with the endothelial cell marker CD31 (Fig 4B). As before, we observed an increase of Nestin, SMA and VEGFR2 markers in tumors as compared to healthy brain tissue, demonstrating the glioblastoma nature of the tumors (Fig 4C-F). We observed a tendency to decreased Nestin- SMA- and VEGFR2-positive cells in tumoral tissue in the *Trf1* deleted brains, which was less evident at the HEP (Fig 4C-F).

### Telomere dysfunction owing to TRF1 abrogation in endothelial cells does not impair glioblastoma growth in a progression model

**Fig 5:**
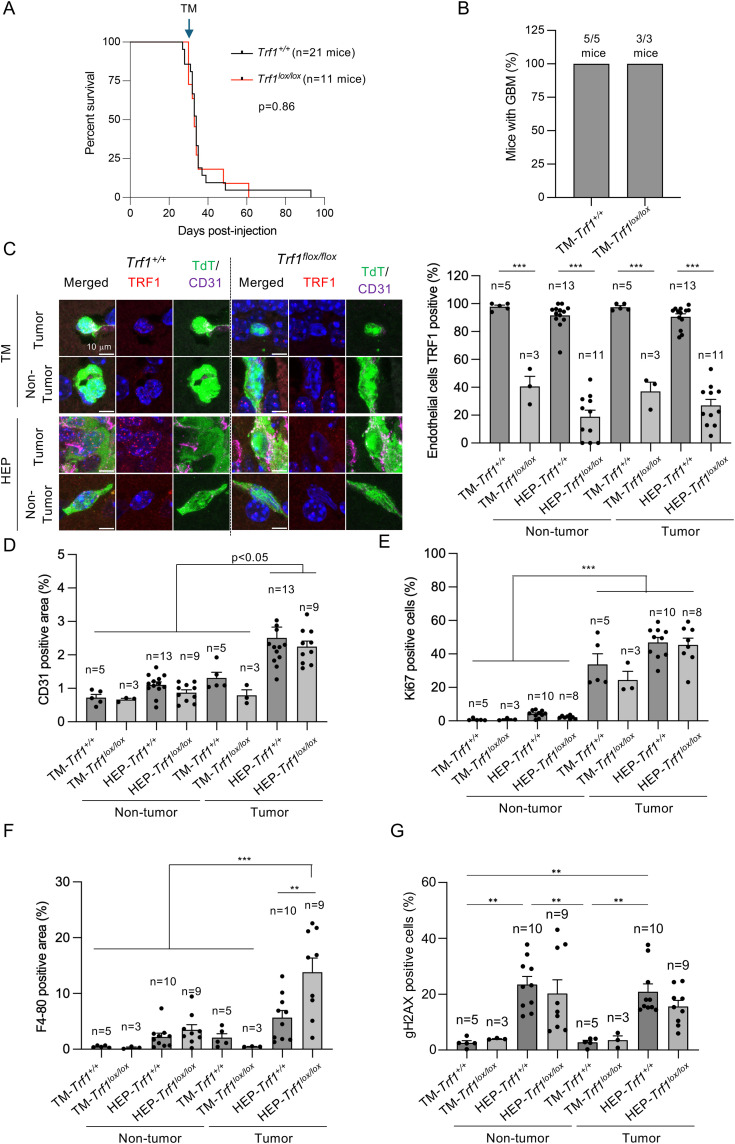
Endothelial *Trf1* deletion during tumor progression does not affect survival or glioblastoma growth. **(A)** Kaplan–Meier survival curves of *Trf1*^*+/+*^ and *Trf1*^*lox/lox*^ mice in the progression model. **(B)** Percentage of mice developing glioblastoma at the time-matched time-point. **(C)** Representative confocal images showing TRF1 (red), tdTomato (green), and CD31 (purple) staining in non-tumoral and tumoral regions, confirming endothelial-specific Trf1 abrogation. Quantification of TRF1-positive endothelial cells is shown on the right. **(D–G)** Quantification of CD31-positive area (D), Ki67-positive cells (E), F4/80-positive cells (F), and γH2AX-positive cells (G) in non-tumoral and tumoral tissues. Data are shown as mean ± SEM; individual dots represent independent mice. Statistical significance was addressed by one-way ANOVA with Tukey’s multiple comparison test, *p < 0.05; **p < 0.01; ***p < 0.001.

We did not see any differences in mouse survival between both cohorts upon *Trf1* deletion in the glioblastoma progression models (Fig 5A). In agreement with this finding, we observed a similar percentage of mice presenting glioblastoma in both mouse cohorts at 4 weeks after post-intracranial RCAS-PDGFB treatment and induction of *Trf1* deletion (Fig 5B). By triple staining with antibodies against TRF1, tdTomato and CD31, we confirmed TRF1 abrogation specifically in endothelial cells both in normal brain tissue and in tumors as indicated by the colocalization of the tomato reporter with the endothelial marker CD31 (Fig 5C).

At the cellular level, we confirmed a tendency to lower vessel formation as indicated by CD31-positive staining in both *Trf1* normal tissue and tumors (Fig 5D).

We did not see changes in cell proliferation as indicated by a similar percentage of Ki67-positive cells in both cohorts in both normal and tumoral tissues, nor changes in global DNA damage as determined by γH2AX in mice with *Trf1* deleted compared to *Trf1* wild-type in endothelial cells. Of notice, we observed a significant increase in the inflammation marked F4-80 in tumors with *Trf1* deleted compared to *Trf1* wild-type (Fig E-G).

As described above for the tumor initiation model, we determined γH2AX and the cell cycle inhibitor p21 specifically in CD31-positive endothelial cells within the tumors. We did not see differences in the amount of DNA damage and cell cycle arrest as determined by γH2AX-positive and p21 positive cells, respectively, in *Trf1* deleted mice compared to *Trf1* wild-type mice (S1 Fig).

**Fig 6:**
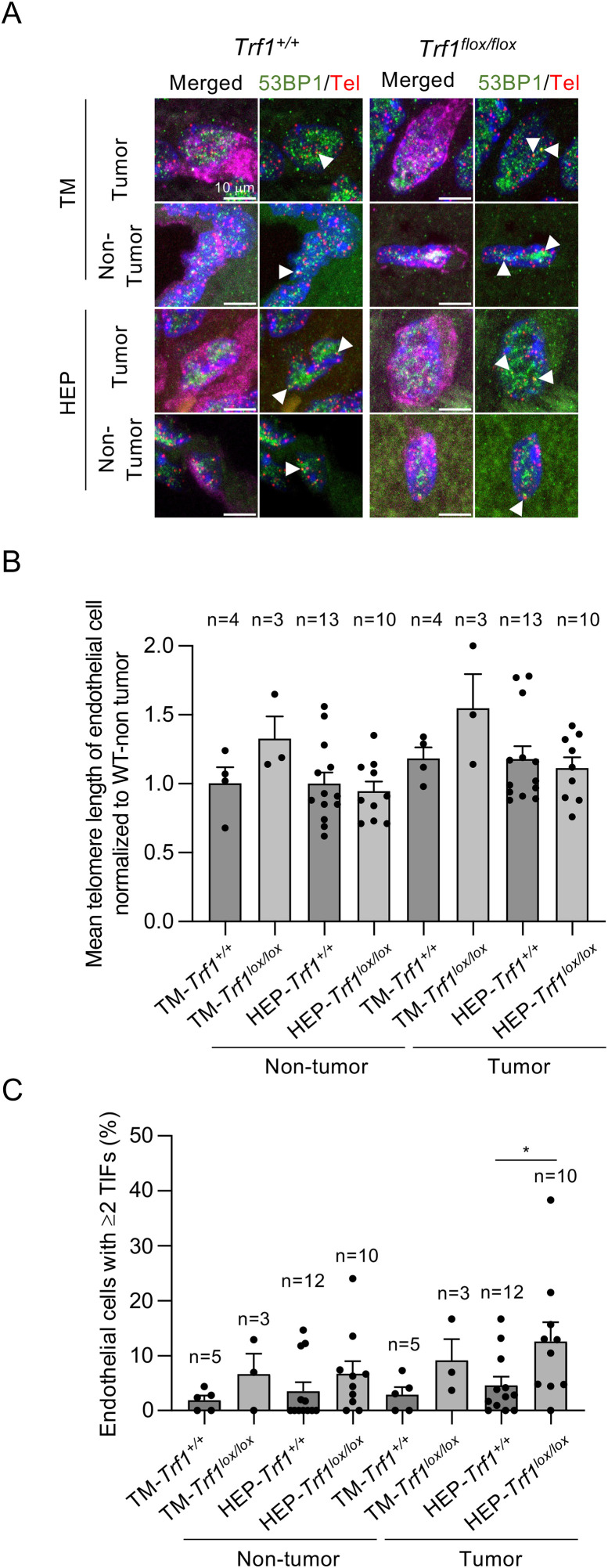
Endothelial *Trf1* deletion does not alter telomere length during tumor progression. **(A)** Representative immuno–telomere Q-FISH images showing 53BP1 (green), telomeres (red), and tdTomato (purple) in endothelial cells from *Trf1*^*+/+*^ and *Trf1*^*lox/lox*^ mice at TM and HEP. Arrowheads indicate telomere dysfunction–induced foci (TIFs). **(B)** Mean telomere length in endothelial cells, normalized to *Trf1*^*+/+*^ non-tumoral tissue. **(C)** Quantification of endothelial cells with >2 TIFs in the progression model. Data are shown as mean ± SEM; individual dots represent independent mice. Statistical significance was addressed by one-way ANOVA with Tukey’s multiple comparison test for telomere length analysis, and unpaired non-parametric t-test for TIFs analysis, *p < 0.05; **p < 0.01; ***p < 0.001.

TRF1 abrogation in endothelial cells did not affect telomere length in the tumors and in the normal brain tissue (Fig 6A,B). As expected, we observed a tendency towards increased DNA damage specifically located at telomeres as indicated by an increase in TIFs in mice with *Trf1* deleted compared to *Trf1* wild-type in endothelial cells (Fig 6C), supporting induction of telomere dysfunction in endothelial cells by TRF1 abrogation.

## Discussion

Previous work has established TRF1 as a critical factor for tumor cell proliferation and survival in aggressive cancers, including glioblastoma and lung cancer in a telomere length–independent manner [19,29]. In particular, TRF1 abrogation in tumor cells induces telomere dysfunction, DNA damage responses, and robust anti-tumor effects. These findings prompted us to investigate whether a similar dependency exists within the tumor microenvironment, specifically in endothelial cells, which play a central role in tumor angiogenesis and vascular remodeling [36].

Glioblastoma is one of the most vascularized solid tumors, and angiogenesis has been considered a critical driver of its initiation and progression [36]. Therefore, endothelial cells and the tumor vasculature have been extensively explored as therapeutic targets. However, reported clinical trials with angiogenesis inhibitors have failed in the clinic [37]. As *Trf1* deletion induces rapid telomere dysfunction, and this has been shown to impair cell viability, in this study, we addressed whether TRF1-mediated telomere protection in endothelial cells had an impact on glioblastoma initiation and progression *in vivo*. We found a tendency to increased telomere induced DNA foci both in the tumor initiation and progression models, which was accompanied by a tendency in decreased CD31-positive cells; however, this was not sufficient to impair tumor growth and tumor associated mortality. The mild effect of *Trf1* deletion on vessel formation could be due to the fact that not all endothelial cells deleted *Trf1*. Nevertheless, our data show that impaired telomere function and decreased CD31-positive cells owing to TRF1 depletion in endothelial cells are not sufficient to stop the initiation and progression of GBM.

These findings suggest that glioblastoma growth is remarkably resilient to impairments in endothelial telomere protection and vascular fitness. One possible explanation is the extensive plasticity of glioblastoma vasculature, which can adapt through alternative angiogenic mechanisms, vessel co-option, or recruitment of non-endothelial stromal components to sustain tumor growth [38].

Our results clearly show that TRF1 depletion in endothelial cells did not affect overall telomere length. This is consistent with previous observations indicating that TRF1 loss primarily compromises telomere protection rather than telomere length maintenance [39,40]. Interestingly, although endothelial *Trf1* deletion induces a trend to increase telomere-associated DNA damage, it did not result in the activation of cell-cycle arrest, apoptosis, or inflammatory responses in vivo. This suggests that endothelial cells within the glioblastoma microenvironment tolerate a certain degree of telomere dysfunction without undergoing cell death, potentially due to compensatory survival pathways. These observations are in line with previous reports showing that anti-angiogenic therapies often fail to produce durable responses in glioblastoma, despite effectively reducing vessel density [41].

Our findings highlight an important distinction between targeting telomere protection in tumor cells versus stromal compartments. While TRF1 is essential for glioblastoma cell proliferation and tumor maintenance [19], its role in endothelial cells is not rate-limiting for tumor growth. This compartment-specific dependency underscores the importance of directly targeting tumor cells rather than cells in the microenvironment, at least in the context of telomere-based therapeutic strategies. From a translational perspective, our results suggest that therapeutic approaches aimed at inhibiting TRF1 or telomere protection are likely to be most effective when directed against tumor cells rather than endothelial cells alone. However, endothelial telomere dysfunction may still contribute to therapeutic responses when combined with additional treatments that limit tumor plasticity.

## Materials and methods

### Mouse models

For the GBM experiments, *Nestin-Tva* [42,43], *Cdkn2a-/-* [44], and *Trf1lox/lox* [39] mouse strains were interbred. This breeding strategy generated *Trf1lox/lox; Nestin-Tva; Cdkn2a-/-* and *Trf1+ / + ; Nestin-Tva; Cdkn2a-/-* models. These mice were subsequently crossed with a strain carrying the tamoxifen-inducible recombinase *TgCdh5-CreERT2; R26-CAG-tdTomato*, expressed in endothelial cells. The final progeny included *Trf1lox/lox; TgCdh5-CreERT2; R26-CAG-tdTomato; Cdkn2a-/-; Nestin-Tva* and *Trf1+ / + ; TgCdh5-CreERT2; R26-CAG-tdTomato; Cdkn2a-/-; Nestin-Tva* mice.

All mice were maintained at the Spanish National Cancer Centre in pathogen-free conditions, under a 12-hour light/dark cycle in temperature-controlled rooms, following the guidelines of the Federation of European Laboratory Animal Science Associations (FELASA). All experimental procedures were approved by the CNIO-ISCIII Ethics Committee and the *Consejería de Medio Ambiente, Administración Local y Ordenación del Territorio* (Comunidad de Madrid). Experiments were conducted in accordance with the international guiding principles for biomedical research involving animals (CIOMS).

Same-sex littermates were assigned to control or experimental groups according to genotype, with the developmental stage specified in each figure and description. Mice had ad libitum access to standard laboratory chow (Harlan Laboratories and Research Diets) and water unless stated otherwise. Euthanasia was performed in CO_2_ chambers upon reaching humane endpoints.

### Generation of mouse models with brain tumors

The RCAS/Tv-a system used in this study has been previously described [42,43]. Adult mice aged 4.5–6.5 weeks received 1 μl injections of DF-1 chicken fibroblasts expressing *RCAS-PDGFB* into the SVZ at a concentration of 200,000 cells/μl. Mice were closely monitored throughout the experiment and euthanized upon showing signs of tumor development. Both male and female mice were used in all procedures.

### Cell culture and transfection

DF1 cells (ATCC) were cultured at 37°C in DMEM (GIBCO) supplemented with 10% FBS (GIBCO). Transfection was performed using the *RCAS-PDGFB* viral plasmid and Fugene 6 reagent (Roche), following the manufacturer’s instructions.

### Immunohistochemistry in tissue sections

Tissues were fixed overnight in 10% formalin (Sigma), embedded in paraffin, and sectioned. Sections were deparaffinized in xylene, rehydrated through graded ethanol, and stained with hematoxylin and eosin for histopathological evaluation.

For immunohistochemistry (IHC), sections were treated with 10 mM sodium citrate buffer (pH 6.5) under pressure for 2 minutes for antigen retrieval. Primary antibodies were applied (see Table 1), and slides were counterstained with hematoxylin and examined by light microscopy. Quantitative image analysis was performed blindly using QuPath [45].

**Table 1:** List of antibodies used in this study.

Antibody	Host species	Source	Ref.
C3 Cleaved Caspase-3 (Asp175)	Rabbit Polyclonal	CELL SIGNALING TECHNOLOGY	9661
CD31 (EPR17259)	Rabbit Monoclonal (EPR17259)	ABCAM	ab182981
E-Cadherin	Mouse Monoclonal (36)	BD BIOSCIENCES	610182
F4/80 Bethyl	Rabbit Monoclonal (BLR209K)	BETHYL	A700-209
Glial Fibrillary Acidic Protein	Rabbit Polyclonal	DAKO	Z0334
Phospho-Histone H2A.X (Ser139)	Mouse Monoclonal (JBW301)	MILLIPORE	05-636
HIF-1 Alpha 343B	Rat Monoclonal (SIMA 343B)	MONOCLONAL ANTIBODIES CORE UNIT	AM (SIMA 343B)
Ki-67 (D3B5)	Rabbit Monoclonal (D3B5)	CELL SIGNALING TECHNOLOGY	12202
Tomato	Goat Polyclonal	Origene	Ab8181
Nestin	Mouse Monoclonal (RAT-401)	MILLIPORE	MAB353
Smooth Muscle Actin (1A4) Flex (B)	Mouse Monoclonal (1A4)	DAKO	IR611
SOX2 (C70B1)	Rabbit Monoclonal (C70B1)	CELL SIGNALING TECHNOLOGY	3728
TRF1		MONOCLONAL ANTIBODIES CORE UNIT	
VEGFR-2	Rabbit Monoclonal (55B11)	CELL SIGNALING TECHNOLOGY	2479

### Immunofluorescence in cells and tissue sections

Tissues were fixed in 10% buffered formalin (Sigma), embedded in paraffin, deparaffinized, and subjected to citrate antigen retrieval. Sections were permeabilized with 0.5% Triton in PBS and blocked with 1% BSA and 10% Australian FBS (GENYCELL) in PBS. Antibodies (listed in the Antibody Table) were incubated overnight in diluents containing background-reducing agents (Invitrogen). Images were acquired using a Leica TCS-SP8 confocal ultraspectral microscope.

Quantification of TRF1 in endothelial cells was performed manually.

### Quantitative fluorescence in situ hybridization (Q-FISH)

For Q-FISH, paraffin-embedded sections were deparaffinized, fixed in 4% formaldehyde, digested with pepsin/HCl, and refixed. Slides were dehydrated (70%, 90%, 100% ethanol) and hybridized with a Cy3-labeled (TTAGGG)_3_ probe for 3 min at 85°C followed by 1 h at room temperature. After washing with 50% formamide and 0.08% TBS–Tween 20, immuno-telomere Q-FISH was performed using the DNA damage marker 53 BP1 (1:500, Novus Biologicals), as described [46]. Confocal imaging was carried out at room temperature using a Leica TCS SP8 microscope with a Plan Apo 63 × , 1.40 NA oil objective. Z-stack projections were analyzed using LAS X software, with DAPI images used for nuclear localization of telomeric signals.

For telomere length analysis, a robust pipeline was implemented using Cellpose [47] for nuclear segmentation and telomere spot detection. The workflow included telomere and nucleus detection, telomere–nucleus pairing, and feature extraction to assess telomere localization. The segmentation model was fine-tuned for this dataset to ensure accurate telomere spot identification. For tdTomato identification, fluorescence intensity distributions were measured within nuclear regions. Thresholds were determined based on mean and standard deviation values. Nuclei with significantly higher tdTomato intensities were classified as positive, and telomere quantification was then performed within these Tomato⁺ nuclei to evaluate telomere localization in endothelial cells.

### Statistical analysis

For comparisons among four independent groups, one-way ANOVA followed by Tukey’s multiple comparisons test was applied. Animal survival was evaluated using Kaplan–Meier analysis with the log-rank (Mantel–Cox) test. All statistical analyses were performed in GraphPad Prism (version 10.1.1).

## Supporting information

S1 Fig Endothelial *Trf1* deletion does not alter DNA damage or cell-cycle arrest in endothelial cells. **(A)** Representative immunohistochemical images showing CD31/γH2AX (blue/purple) and CD31/p21 (blue/purple) staining in endothelial cells from *Trf1*^*+/+*^ and *Trf1*^*lox/lox*^ mice. Arrows indicate double positive endothelial cells. **(B, C)** Quantification of CD31/γH2AX double positive (B) and CD31/p21 double positive (C) endothelial cells. Data are shown as mean ± SEM; individual dots represent independent mice. Statistical significance was addressed by one-way ANOVA with Tukey’s multiple comparison test.
(PDF)
