# Supplementary material for: Targeting telomeres in brain vasculature does not impede initiation and progression of glioblastoma
Source: PLoS One. 2026 Aug 12;21(8):e0355168. doi: 10.1371/journal.pone.0355168 (PMC13465819; doi:10.1371/journal.pone.0355168)

Supplementary Fig. 1

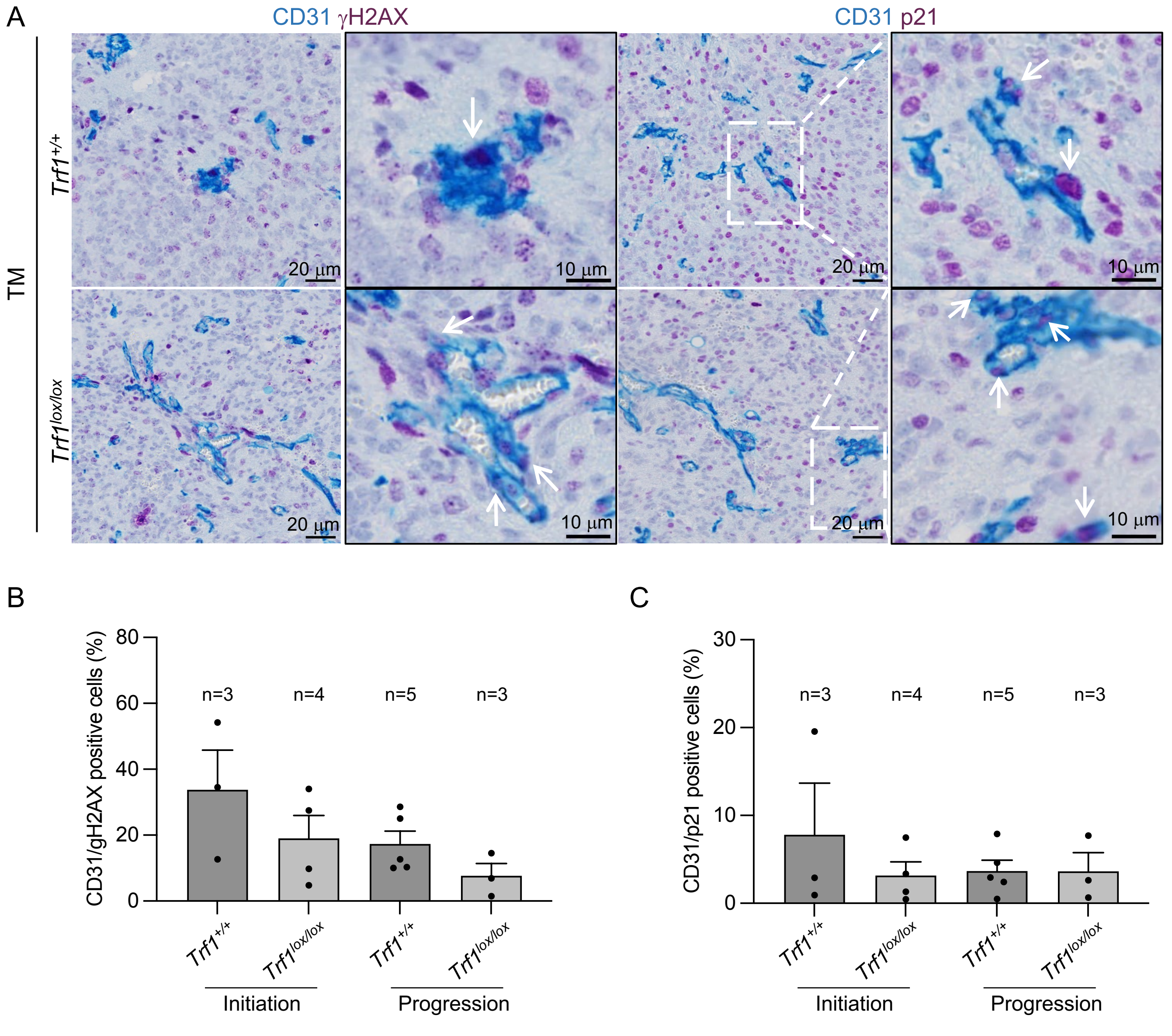

Supplement: Supplementary file 1 [file pone.0355168.s001.pdf]
