# Supplementary material for: Targeting telomeres in brain vasculature does not impede initiation and progression of glioblastoma
Source: PLoS One. 2026 Aug 12;21(8):e0355168. doi: 10.1371/journal.pone.0355168 (PMC13465819; doi:10.1371/journal.pone.0355168)
Supplement: Supplementary file 2 [file pone.0355168.s003.docx]

**Point-by-point response to reviewers’ comments**

**Detailed answers to Reviewer 1 Comments to Author**

1. Is the manuscript technically sound, and do the data support the conclusions? The manuscript must describe a technically sound piece of scientific research with data that supports the conclusions. Experiments must have been conducted rigorously, with appropriate controls, replication, and sample sizes. The conclusions must be drawn appropriately based on the data presented.

Reviewer #1: Yes

**Authors:** We thank the reviewer for his/her thorough review of our manuscript and for considering that our work “Targeting telomeres in brain vasculature does not impede initiation and progression of glioblastoma” is technically appropriate and well conducted.

2. Has the statistical analysis been performed appropriately and rigorously?

Reviewer #1: No

**Authors:** We appreciate the reviewer’s comment regarding the statistical analysis. Our data was analyzed using one-way ANOVA followed by Tukey’s multiple comparisons test. Accordingly, we revised the “Statistical Analysis” section in the Materials and Methods section to clearly describe the statistical analysis performed and to correct the previous description. Additionally, following the reviewer recommendation, we revised the statistical comparisons presented in the manuscript and removed comparisons that are not biologically relevant to the main conclusions of the study, such as: TM Trf1+/+ versus HEP Trf1lox/lox; TM Trf1lox/lox vs HEP Trf1+/+.

We agree that these comparisons between different genotypes across distinct experimental cohorts do not provide meaningful information for the interpretation of our results.

3. Have the authors made all data underlying the findings in their manuscript fully available? The PLOS Data policy requires authors to make all data underlying the findings described in their manuscript fully available without restriction, with rare exception (please refer to the Data Availability Statement in the manuscript PDF file). The data should be provided as part of the manuscript or its supporting information, or deposited to a public repository. For example, in addition to summary statistics, the data points behind means, medians and variance measures should be available. If there are restrictions on publicly sharing data—e.g. participant privacy or use of data from a third party—those must be specified.

Reviewer #1: Yes

**Authors:** We thank the reviewer for the positive evaluation of the clarity, readability, and overall presentation of the manuscript.

4. Is the manuscript presented in an intelligible fashion and written in standard English? PLOS ONE does not copyedit accepted manuscripts, so the language in submitted articles must be clear, correct, and unambiguous. Any typographical or grammatical errors should be corrected at revision, so please note any specific errors here.

Reviewer #1: Yes

**Authors:** We thank the reviewer for considering the manuscript to be clearly written. We appreciate the positive evaluation of the overall clarity and readability of our work.

5. Review Comments to the Author Please use the space provided to explain your answers to the questions above. You may also include additional comments for the author, including concerns about dual publication, research ethics, or publication ethics. (Please upload your review as an attachment if it exceeds 20,000 characters)

Reviewer #1: Abrogation or inhibition of the TRF1 shelterin telomere protein in a GBM model delays tumor growth and progression. The goal was to determine whether Trf1 deletion only in endothelial cells, has an impact on GBM growth and progression. Trf1 deletion was associated to a decrease in CD31-positive cells in GBM tumors and an increase in induced DNA damage foci in endothelial cells. Yet, Trf1 deletion was not sufficient to delay tumor growth and progression.

This work is interesting and well-conducted. My only concern is that the effect of Trf1 surexpression in endothelial cells have not been assessed.

**Authors:** We appreciate the reviewer’s comment. The suggested approach is indeed interesting, as the role of TRF1 in endothelial cells could potentially be explored through both upregulation and downregulation strategies. In the present study, however, our main objective was to evaluate whether endothelial-specific deletion of Trf1 could impair glioblastoma by affecting tumor-associated vasculature.

TRF1 overexpression has been associated with several cancer types, including glioblastoma. Consequently, TRF1 inhibition has been proposed as a potential anti-tumoral strategy due to its association with telomere dysfunction, DNA damage accumulation, and impaired cell viability. Given the strong vascular component of glioblastoma, we focused specifically on Trf1 deletion in endothelial cells to determine whether targeting telomere protection in the tumor vasculature could limit tumor growth.

We agree that assessing the effects of TRF1 overexpression in endothelial cells could provide additional insight into the role of telomere protection in the tumor microenvironment and may represent an interesting avenue for future studies.

**Minor remarks:**

1. Abstract: the last sentence “These findings question an important role of vessel fitness in tumor initiation and progression.” is not perfectly clear.

**Authors:** We agree with the reviewer that the sentence was not sufficiently clear. We intended to suggest that, although vessel architecture and function are important components of glioblastoma, TRF1 in endothelial cells does not appear to be a major limiting factor for tumor initiation and progression in our PDGFB-driven glioblastoma model.

To clarify this point, we have modified the sentence in the Abstract from:

“**These findings question an important role of vessel fitness in tumor initiation and progression**.”

to:

“**These findings suggest that TRF1-dependent telomere protection in endothelial cells is not a major limiting factor for PDGF-driven GBM initiation and progression**”.

2. Glioblastoma multiform is a term that is not used anymore since the 2021 WHO classification has been published.

**Authors:** We agree with the reviewer and apologize for the inaccurate terminology. As “glioblastoma” is the correct term under the current WHO classification, we have revised the manuscript to replace “glioblastoma multiforme” with “glioblastoma”. The abbreviation GBM has been maintained, as it remains widely used in the field and throughout the current literature.

3. Introduction: “the median survival has not improved in the last 50 years,”. This is false as the Stupp protocol and TTfields significantly improved the OS.

**Authors:** We thank the reviewer for this important observation and agree that the original statement was inaccurate. Although glioblastoma prognosis remains poor, relevant therapeutic advances have been achieved and should be considered, including the Stupp protocol (the use of temozolomide following radiotherapy) and Tumor Treating Fields (TTFields), both of which have contributed to improvements in overall survival. Accordingly, we have included appropriate citations supporting these therapeutic approaches.

To address this issue, we have revised the sentence:

“**Despite all the advances in the molecular characterization of glioblastoma, the median survival has not improved in the last 50 years, being only 14-16 months**”

to:

“**Despite the advances in the molecular characterization of glioblastoma and improvements in therapeutic approaches, including the Stupp protocol and Tumor Treating Fields (TTFields), patient prognosis remains poor, with a median survival of approximately 14–16 months**”-

4. Introduction: to exemplify GB heterogeneity, stronger paper should be cited

**Authors:** We thank the reviewer for this observation. It is true that, in the Introduction section, the sentence: “While these approaches show promise, the immunosuppressive tumor microenvironment and high heterogeneity of GBM remain significant challenges” lacked appropriate reference support regarding glioblastoma heterogeneity. Accordingly, we have revised the literature and incorporated appropriate citations in support of this statement.

5. What does “as well as for the initiation of tumor cells in the brain” means?

**Authors:** We appreciate the reviewer’s comment and agree that the original sentence was not sufficiently clear and could be misleading.

In the original sentence: “**Among these cell populations, endothelial cells are of particular importance as they allow for the high vascularization of these tumors, as well as for the initiation of tumor cells in the brain**” we intended to express that endothelial cells play an important role in glioblastoma progression not only by contributing to tumor vascularization, but also through interactions with multiple components of the tumor microenvironment, including tumor cells. These interactions ultimately contribute to tumor initiation and progression.

To improve clarity, we have modified the sentence as follows:

“**Among these cell populations, endothelial cells are of particular importance as they contribute to the high vascularization of these tumors, and support the tumor microenvironment by connecting tumor cells with infiltrating immune cells, glioma stem cells, and the extracellular matrix, which drives tumor progression**”.

6. In some figure, irrelevant comparisons were performed (ex: 1D, 2E, 4D, 5G…)

**Authors:** We thank the reviewer for this comment, and agree that some of the original comparisons were not informative for the main objective of the study. After revising the statistical analysis, we concluded that the most biologically relevant comparisons, and therefore the ones prioritized in our analysis, are:

1. Comparisons between Trf1+/+ and Trf1lox/lox mice within the same cohort (TM or HEP). Through these comparisons, we sought to assess whether endothelial-specific deletion of Trf1 induces changes relative to the wild-type condition within each cohort.

2. Comparisons between TM and HEP conditions within the same genotype (Trf1+/+ or Trf1lox/lox). Through these comparisons, we sought to evaluate how the analyzed markers change between the TM and HEP time points within each genotype.

Therefore, we removed comparisons that were not directly relevant to the biological questions addressed in this study and that were not essential for supporting the conclusions of the manuscript. **The following figures are corrected: Figure 1.D, 2.E, 4 D, 5 G**.

7. There are some typos. “Defficient” for instance. There are some grammatical mistakes as well (for instance: “therapeutics strategies against GBM has been tried”).

**Authors:** We sincerely thank the reviewer for identifying these grammatical and typographical errors. Accordingly, we have thoroughly revised the manuscript and corrected the identified mistakes throughout the test, including typographical and grammatical errors (e.g. “Defficient” corrected to “deficient” or “therapeutics strategies against GBM has been tried” corrected to “therapeutic strategies against GBM have been tried”).

In addition, we have revised terminology for consistency (e.g., replacing “glioblastoma multiforme” with “glioblastoma” and standardizing the use of “tdTomato”), as well as punctuation and stylistic issues to improve clarity and readability.

“TIF” has to be defined before being used.

**Authors:** We thank the reviewer for this suggestion. Accordingly, we have defined the term before its first use in the manuscript. The sentence was:

“**As expected, we observed a tendency towards increased DNA damage specifically located at telomeres as indicated by increased telomere DNA damage foci or TIFs in mice with Trf1 deleted compared to Trf1 wild-type in endothelial cells. TIFs are detected by colocalization between telomeres and the 53BP1 protein (Fig. 3B,C)**.”

and has been revised to:

“**Next, we sought to assess telomeric DNA damage, as indicated by telomere dysfunction-induced foci (TIFs). TIFs were detected by colocalization between telomeres and the DNA damage marker 53BP1 protein. As expected, we observed a tendency towards increased DNA damage specifically located at telomeres, as indicated by increased TIFs in mice with Trf1 deleted compared to Trf1 wild-type in endothelial cells (Fig 3B,C)**”.

6. PLOS authors have the option to publish the peer review history of their article (what does this mean?). If published, this will include your full peer review and any attached files. If you choose “no”, your identity will remain anonymous but your review may still be made public. Do you want your identity to be public for this peer review? For information about this choice, including consent withdrawal, please see our Privacy Policy.

Reviewer #1: No
