# Supplementary material for: Targeting telomeres in brain vasculature does not impede initiation and progression of glioblastoma
Source: PLoS One. 2026 Aug 12;21(8):e0355168. doi: 10.1371/journal.pone.0355168 (PMC13465819; doi:10.1371/journal.pone.0355168)
Supplement: Peer review history

# Peer review: Decision Letter 0

**DOI:** 10.1371/journal.pone.0355168.r001

**Authors:** Gabriele Saretzki

21 May 2026

Dear Dr. Blasco,

Thank you for submitting your manuscript to PLOS ONE. After careful consideration, we feel that it has merit but does not fully meet PLOS ONE’s publication criteria as it currently stands. Therefore, we invite you to submit a revised version of the manuscript that addresses the points raised during the review process.

Please submit your revised manuscript by Jul 05 2026 11:59PM. If you will need more time than this to complete your revisions, please reply to this message or contact the journal office at plosone@plos.org. When you're ready to submit your revision, log on to https://www.editorialmanager.com/pone/ and select the 'Submissions Needing Revision' folder to locate your manuscript file.

- A letter that responds to each point raised by the academic editor and reviewer(s). You should upload this letter as a separate file labeled 'Response to Reviewers'.
- A marked-up copy of your manuscript that highlights changes made to the original version. You should upload this as a separate file labeled 'Revised Manuscript with Track Changes'.
- An unmarked version of your revised paper without tracked changes. You should upload this as a separate file labeled 'Manuscript'.

If you would like to make changes to your financial disclosure, please include your updated statement in your cover letter. Guidelines for resubmitting your figure files are available below the reviewer comments at the end of this letter.

If applicable, we recommend that you deposit your laboratory protocols in protocols.io to enhance the reproducibility of your results. Protocols.io assigns your protocol its own identifier (DOI) so that it can be cited independently in the future. For instructions see: https://journals.plos.org/plosone/s/submission-guidelines#loc-laboratory-protocols. Additionally, PLOS ONE offers an option for publishing peer-reviewed Lab Protocol articles, which describe protocols hosted on protocols.io. Read more information on sharing protocols at https://plos.org/protocols?utm_medium=editorial-email&utm_source=authorletters&utm_campaign=protocols.

As the corresponding author, your ORCID iD is verified in the submission system and will appear in the published article. PLOS supports the use of ORCID, and we encourage all coauthors to register for an ORCID iD and use it as well. Please encourage your coauthors to verify their ORCID iD within the submission system before final acceptance, as unverified ORCID iDs will not appear in the published article. *Only*  the individual author can complete the verification step; PLOS staff *cannot*  verify ORCID iDs on behalf of authors.

We look forward to receiving your revised manuscript.

Kind regards,

Gabriele Saretzki, PhD

Academic Editor

PLOS One

Journal Requirements:

When submitting your revision, we need you to address these additional requirements.

1. Please ensure that your manuscript meets PLOS ONE's style requirements, including those for file naming. The PLOS ONE style templates can be found at

https://journals.plos.org/plosone/s/file?id=wjVg/PLOSOne_formatting_sample_main_body.pdf and

https://journals.plos.org/plosone/s/file?id=ba62/PLOSOne_formatting_sample_title_authors_affiliations.pdf

2. Thank you for stating the following in the Acknowledgments Section of your manuscript:

“Research in the Blasco lab is funded by the European Union, project ERC-AvG Shelterins (GA882385), Horizon 2020 Program. We would like to thank to Histopathology Unit (CNIO) for their assistance. The founders had no role in study design, data collection and analysis, decision to publish, or preparation of the manuscript.”

We note that you have provided funding information that is not currently declared in your Funding Statement. However, funding information should not appear in the Acknowledgments section or other areas of your manuscript. We will only publish funding information present in the Funding Statement section of the online submission form.

Please remove any funding-related text from the manuscript and let us know how you would like to update your Funding Statement. Currently, your Funding Statement reads as follows:

“European Union, project ERC-AvG Shelterins (GA882385), Horizon 2020 Program”

Please include your amended statements within your cover letter; we will change the online submission form on your behalf.

3. Thank you for stating the following financial disclosure:

“European Union, project ERC-AvG Shelterins (GA882385), Horizon 2020 Program”

Please state what role the funders took in the study.  If the funders had no role, please state: "The funders had no role in study design, data collection and analysis, decision to publish, or preparation of the manuscript."

If this statement is not correct you must amend it as needed.

Please include this amended Role of Funder statement in your cover letter; we will change the online submission form on your behalf.

4. We note that your Data Availability Statement is currently as follows: “All relevant data are within the manuscript and its Supporting Information files.”

Please confirm at this time whether or not your submission contains all raw data required to replicate the results of your study. Authors must share the “minimal data set” for their submission. PLOS defines the minimal data set to consist of the data required to replicate all study findings reported in the article, as well as related metadata and methods (https://journals.plos.org/plosone/s/data-availability#loc-minimal-data-set-definition).

For example, authors should submit the following data:

- The values behind the means, standard deviations and other measures reported;

- The values used to build graphs;

- The points extracted from images for analysis.

Authors do not need to submit their entire data set if only a portion of the data was used in the reported study.

If your submission does not contain these data, please either upload them as Supporting Information files or deposit them to a stable, public repository and provide us with the relevant URLs, DOIs, or accession numbers. For a list of recommended repositories, please see https://journals.plos.org/plosone/s/recommended-repositories.

If there are ethical or legal restrictions on sharing a de-identified data set, please explain them in detail (e.g., data contain potentially sensitive information, data are owned by a third-party organization, etc.) and who has imposed them (e.g., an ethics committee). Please also provide contact information for a data access committee, ethics committee, or other institutional body to which data requests may be sent. If data are owned by a third party, please indicate how others may request data access.

5. Please include a separate caption for each figure in your manuscript.

6. We notice that your supplementary figures are uploaded with the file type 'Figure'. Please amend the file type to 'Supporting Information'. Please ensure that each Supporting Information file has a legend listed in the manuscript after the references list.

7. If the reviewer comments include a recommendation to cite specific previously published works, please review and evaluate these publications to determine whether they are relevant and should be cited. There is no requirement to cite these works unless the editor has indicated otherwise.

Additional Editor Comments:

Please follow the suggestions and comments from the reviewer in order to improve your study.

[Note: HTML markup is below. Please do not edit.]

Reviewers' comments:

Reviewer's Responses to Questions

**Comments to the Author**

1. Is the manuscript technically sound, and do the data support the conclusions?

Reviewer #1: Yes

**********

2. Has the statistical analysis been performed appropriately and rigorously?

Reviewer #1: No

**********

3. Have the authors made all data underlying the findings in their manuscript fully available?

The PLOS Data policy

Reviewer #1: Yes

**********

4. Is the manuscript presented in an intelligible fashion and written in standard English?

Reviewer #1: Yes

**********

Reviewer #1: Abrogation or inhibition of the TRF1 shelterin telomere protein in a GBM model delays tumor growth and progression. The goal was to determine whether Trf1 deletion only in endothelial cells, has an impact on GBM growth and progression. Trf1 deletion was associated to a decrease in CD31-positive cells in GBM tumors and an increase in induced DNA damage foci in endothelial cells. Yet, Trf1 deletion was not sufficient to delay tumor growth and progression.

This work is interesting and well-conducted. My only concern is that the effect of Trf1 surexpression in endothelial cells have not been assessed.

Minor remarks:

1. Abstract: the last sentence “These findings question an important role of vessel fitness in tumor initiation and progression.” is not perfectly clear.

2. Glioblastoma multiform is a term that is not used anymore since the 2021 WHO classification has been published.

3. Introduction: “the median survival has not improved in the last 50 years,”. This is false as the Stupp protocol and TTfields significantly improved the OS.

4. Introduction: to exemplify GB heterogeneity, stronger paper should be cited

5. What does “as well as for the initiation of tumor cells in the brain” means?

6. In some figure, irrelevant comparisons were performed (ex: 1D, 2E, 4D, 5G…)

7. There are some typos. “Defficient” for instance. There are some grammatical mistakes as well (for instance: “therapeutics strategies against GBM has been tried”). “TIF” has to be defined before being used.

**********

what does this mean?). If published, this will include your full peer review and any attached files.

If you choose “no”, your identity will remain anonymous but your review may still be made public.

**Do you want your identity to be public for this peer review?**  For information about this choice, including consent withdrawal, please see our Privacy Policy

Reviewer #1: No

**********

[NOTE: If reviewer comments were submitted as an attachment file, they will be attached to this email and accessible via the submission site. Please log into your account, locate the manuscript record, and check for the action link "View Attachments". If this link does not appear, there are no attachment files.]

To ensure your figures meet our technical requirements, please review our figure guidelines: https://journals.plos.org/plosone/s/figures

You may also use PLOS’s free figure tool, NAAS, to help you prepare publication quality figures: https://journals.plos.org/plosone/s/figures#loc-tools-for-figure-preparation.

NAAS will assess whether your figures meet our technical requirements by comparing each figure against our figure specifications.

# Author comment: Author response to Decision Letter 1

**DOI:** 10.1371/journal.pone.0355168.r002

14 Jul 2026

Point-by-point response to reviewers’ comments

Detailed answers to Reviewer 1 Comments to Author

1. Is the manuscript technically sound, and do the data support the conclusions? The manuscript must describe a technically sound piece of scientific research with data that supports the conclusions. Experiments must have been conducted rigorously, with appropriate controls, replication, and sample sizes. The conclusions must be drawn appropriately based on the data presented.

Reviewer #1: Yes

Authors: We thank the reviewer for his/her thorough review of our manuscript and for considering that our work “Targeting telomeres in brain vasculature does not impede initiation and progression of glioblastoma” is technically appropriate and well conducted.

2. Has the statistical analysis been performed appropriately and rigorously?

Reviewer #1: No

Authors: We appreciate the reviewer’s comment regarding the statistical analysis. Our data was analyzed using one-way ANOVA followed by Tukey’s multiple comparisons test. Accordingly, we revised the “Statistical Analysis” section in the Materials and Methods section to clearly describe the statistical analysis performed and to correct the previous description. Additionally, following the reviewer recommendation, we revised the statistical comparisons presented in the manuscript and removed comparisons that are not biologically relevant to the main conclusions of the study, such as: TM Trf1+/+ versus HEP Trf1lox/lox; TM Trf1lox/lox vs HEP Trf1+/+.

We agree that these comparisons between different genotypes across distinct experimental cohorts do not provide meaningful information for the interpretation of our results.

3. Have the authors made all data underlying the findings in their manuscript fully available? The PLOS Data policy requires authors to make all data underlying the findings described in their manuscript fully available without restriction, with rare exception (please refer to the Data Availability Statement in the manuscript PDF file). The data should be provided as part of the manuscript or its supporting information, or deposited to a public repository. For example, in addition to summary statistics, the data points behind means, medians and variance measures should be available. If there are restrictions on publicly sharing data—e.g. participant privacy or use of data from a third party—those must be specified.

Reviewer #1: Yes

Authors: We thank the reviewer for the positive evaluation of the clarity, readability, and overall presentation of the manuscript.

4. Is the manuscript presented in an intelligible fashion and written in standard English? PLOS ONE does not copyedit accepted manuscripts, so the language in submitted articles must be clear, correct, and unambiguous. Any typographical or grammatical errors should be corrected at revision, so please note any specific errors here.

Reviewer #1: Yes

Authors: We thank the reviewer for considering the manuscript to be clearly written. We appreciate the positive evaluation of the overall clarity and readability of our work.

5. Review Comments to the Author Please use the space provided to explain your answers to the questions above. You may also include additional comments for the author, including concerns about dual publication, research ethics, or publication ethics. (Please upload your review as an attachment if it exceeds 20,000 characters)

Reviewer #1: Abrogation or inhibition of the TRF1 shelterin telomere protein in a GBM model delays tumor growth and progression. The goal was to determine whether Trf1 deletion only in endothelial cells, has an impact on GBM growth and progression. Trf1 deletion was associated to a decrease in CD31-positive cells in GBM tumors and an increase in induced DNA damage foci in endothelial cells. Yet, Trf1 deletion was not sufficient to delay tumor growth and progression.

This work is interesting and well-conducted. My only concern is that the effect of Trf1 surexpression in endothelial cells have not been assessed.

Authors: We appreciate the reviewer’s comment. The suggested approach is indeed interesting, as the role of TRF1 in endothelial cells could potentially be explored through both upregulation and downregulation strategies. In the present study, however, our main objective was to evaluate whether endothelial-specific deletion of Trf1 could impair glioblastoma by affecting tumor-associated vasculature.

TRF1 overexpression has been associated with several cancer types, including glioblastoma. Consequently, TRF1 inhibition has been proposed as a potential anti-tumoral strategy due to its association with telomere dysfunction, DNA damage accumulation, and impaired cell viability. Given the strong vascular component of glioblastoma, we focused specifically on Trf1 deletion in endothelial cells to determine whether targeting telomere protection in the tumor vasculature could limit tumor growth.

We agree that assessing the effects of TRF1 overexpression in endothelial cells could provide additional insight into the role of telomere protection in the tumor microenvironment and may represent an interesting avenue for future studies.

Minor remarks:

1. Abstract: the last sentence “These findings question an important role of vessel fitness in tumor initiation and progression.” is not perfectly clear.

Authors: We agree with the reviewer that the sentence was not sufficiently clear. We intended to suggest that, although vessel architecture and function are important components of glioblastoma, TRF1 in endothelial cells does not appear to be a major limiting factor for tumor initiation and progression in our PDGFB-driven glioblastoma model.

To clarify this point, we have modified the sentence in the Abstract from:

“These findings question an important role of vessel fitness in tumor initiation and progression.”

to:

“These findings suggest that TRF1-dependent telomere protection in endothelial cells is not a major limiting factor for PDGF-driven GBM initiation and progression”.

2. Glioblastoma multiform is a term that is not used anymore since the 2021 WHO classification has been published.

Authors: We agree with the reviewer and apologize for the inaccurate terminology. As “glioblastoma” is the correct term under the current WHO classification, we have revised the manuscript to replace “glioblastoma multiforme” with “glioblastoma”. The abbreviation GBM has been maintained, as it remains widely used in the field and throughout the current literature.

3. Introduction: “the median survival has not improved in the last 50 years,”. This is false as the Stupp protocol and TTfields significantly improved the OS.

Authors: We thank the reviewer for this important observation and agree that the original statement was inaccurate. Although glioblastoma prognosis remains poor, relevant therapeutic advances have been achieved and should be considered, including the Stupp protocol (the use of temozolomide following radiotherapy) and Tumor Treating Fields (TTFields), both of which have contributed to improvements in overall survival. Accordingly, we have included appropriate citations supporting these therapeutic approaches.

To address this issue, we have revised the sentence:

“Despite all the advances in the molecular characterization of glioblastoma, the median survival has not improved in the last 50 years, being only 14-16 months”

to:

“Despite the advances in the molecular characterization of glioblastoma and improvements in therapeutic approaches, including the Stupp protocol and Tumor Treating Fields (TTFields), patient prognosis remains poor, with a median survival of approximately 14–16 months”-

4. Introduction: to exemplify GB heterogeneity, stronger paper should be cited

Authors: We thank the reviewer for this observation. It is true that, in the Introduction section, the sentence: “While these approaches show promise, the immunosuppressive tumor microenvironment and high heterogeneity of GBM remain significant challenges” lacked appropriate reference support regarding glioblastoma heterogeneity. Accordingly, we have revised the literature and incorporated appropriate citations in support of this statement.

5. What does “as well as for the initiation of tumor cells in the brain” means?

Authors: We appreciate the reviewer’s comment and agree that the original sentence was not sufficiently clear and could be misleading.

In the original sentence: “Among these cell populations, endothelial cells are of particular importance as they allow for the high vascularization of these tumors, as well as for the initiation of tumor cells in the brain” we intended to express that endothelial cells play an important role in glioblastoma progression not only by contributing to tumor vascularization, but also through interactions with multiple components of the tumor microenvironment, including tumor cells. These interactions ultimately contribute to tumor initiation and progression.

To improve clarity, we have modified the sentence as follows:

“Among these cell populations, endothelial cells are of particular importance as they contribute to the high vascularization of these tumors, and support the tumor microenvironment by connecting tumor cells with infiltrating immune cells, glioma stem cells, and the extracellular matrix, which drives tumor progression”.

6. In some figure, irrelevant comparisons were performed (ex: 1D, 2E, 4D, 5G…)

Authors: We thank the reviewer for this comment, and agree that some of the original comparisons were not informative for the main objective of the study. After revising the statistical analysis, we concluded that the most biologically relevant comparisons, and therefore the ones prioritized in our analysis, are:

1. Comparisons between Trf1+/+ and Trf1lox/lox mice within the same cohort (TM or HEP). Through these comparisons, we sought to assess whether endothelial-specific deletion of Trf1 induces changes relative to the wild-type condition within each cohort.

2. Comparisons between TM and HEP conditions within the same genotype (Trf1+/+ or Trf1lox/lox). Through these comparisons, we sought to evaluate how the analyzed markers change between the TM and HEP time points within each genotype.

Therefore, we removed comparisons that were not directly relevant to the biological questions addressed in this study and that were not essential for supporting the conclusions of the manuscript. The following figures are corrected: Figure 1.D, 2.E, 4 D, 5 G.

7. There are some typos. “Defficient” for instance. There are some grammatical mistakes as well (for instance: “therapeutics strategies against GBM has been tried”).

Authors: We sincerely thank the reviewer for identifying these grammatical and typographical errors. Accordingly, we have thoroughly revised the manuscript and corrected the identified mistakes throughout the test, including typographical and grammatical errors (e.g. “Defficient” corrected to “deficient” or “therapeutics strategies against GBM has been tried” corrected to “therapeutic strategies against GBM have been tried”).

In addition, we have revised terminology for consistency (e.g., replacing “glioblastoma multiforme” with “glioblastoma” and standardizing the use of “tdTomato”), as well as punctuation and stylistic issues to improve clarity and readability.

“TIF” has to be defined before being used.

Authors: We thank the reviewer for this suggestion. Accordingly, we have defined the term before its first use in the manuscript. The sentence was:

“As expected, we observed a tendency towards increased DNA damage specifically located at telomeres as indicated by increased telomere DNA damage foci or TIFs in mice with Trf1 deleted compared to Trf1 wild-type in endothelial cells. TIFs are detected by colocalization between telomeres and the 53BP1 protein (Fig. 3B,C).”

and has been revised to:

“Next, we sought to assess telomeric DNA damage, as indicated by telomere dysfunction-induced foci (TIFs). TIFs were detected by colocalization between telomeres and the DNA damage marker 53BP1 protein. As expected, we observed a tendency towards increased DNA damage specifically located at telomeres, as indicated by increased TIFs in mice with Trf1 deleted compared to Trf1 wild-type in endothelial cells (Fig 3B,C)”.

6. PLOS authors have the option to publish the peer review history of their article (what does this mean?). If published, this will include your full peer review and any attached files. If you choose “no”, your identity will remain anonymous but your review may still be made public. Do you want your identity to be public for this peer review? For information about this choice, including consent withdrawal, please see our Privacy Policy.

Reviewer #1: No

Response to editors comments:

We appreciate the editor's comments regarding the manuscript and associated data. In aggreement with these comments:

- We have removed all funding-related information from the Acknowledgments section of the manuscript and included it in the Funding Statement section as follows: “Research in the Blasco laboratory is funded by the European Union through the ERC Advanced Grant Shelterins (GA882385) under the Horizon 2020 Programme. The funders had no role in study design, data collection and analysis, decision to publish, or preparation of the manuscript.”

-We have included an Excel file containing the data underlying the graphs presented in the manuscript. The file is organized into separate worksheets corresponding to each figure and includes the values used to build the graphs.

We appreciate both reviewer's and editor's comments and hope that the revised version of the manuscript meets PLOS ONE's publication criteria.

Attachment Submitted filename: SANCHEZ_Point by point response to reviewers.docx

# Editor report: Decision Letter 1

**DOI:** 10.1371/journal.pone.0355168.r003

**Authors:** Gabriele Saretzki

20 Jul 2026

Targeting telomeres in brain vasculature does not impede initiation and progression of glioblastoma

PONE-D-26-08221R1

Dear Prof. Blasco,

We’re pleased to inform you that your manuscript has been judged scientifically suitable for publication and will be formally accepted for publication once it meets all outstanding technical requirements.

Within one week, you’ll receive an e-mail detailing the required amendments. When these have been addressed, you’ll receive a formal acceptance letter and your manuscript will be scheduled for publication.

An invoice will be generated when your article is formally accepted. Please note, if your institution has a publishing partnership with PLOS and your article meets the relevant criteria, all or part of your publication costs will be covered. Please make sure your user information is up-to-date by logging into Editorial Manager at Editorial Manager® and clicking the ‘Update My Information' link at the top of the page. For questions related to billing, please contact billing support.

If your institution or institutions have a press office, please notify them about your upcoming paper to help maximize its impact. If they’ll be preparing press materials, please inform our press team as soon as possible -- no later than 48 hours after receiving the formal acceptance. Your manuscript will remain under strict press embargo until 2 pm Eastern Time on the date of publication. For more information, please contact onepress@plos.org.

Kind regards,

Gabriele Saretzki, PhD

Academic Editor

PLOS One

Additional Editor Comments (optional):

The authors have addressed the comments from the reviewer and improved their ms.

Reviewers' comments:

# Editor report: Acceptance letter

**DOI:** 10.1371/journal.pone.0355168.r004

**Authors:** Gabriele Saretzki

PONE-D-26-08221R1

PLOS One

Dear Dr. Blasco,

I'm pleased to inform you that your manuscript has been deemed suitable for publication in PLOS One. Congratulations! Your manuscript is now being handed over to our production team.

At this stage, our production department will prepare your paper for publication. This includes ensuring the following:

* All references, tables, and figures are properly cited

* All relevant supporting information is included in the manuscript submission,

* There are no issues that prevent the paper from being properly typeset

You will receive further instructions from the production team, including instructions on how to review your proof when it is ready. Please keep in mind that we are working through a large volume of accepted articles, so please give us a few days to review your paper and let you know the next and final steps.

Lastly, if your institution or institutions have a press office, please let them know about your upcoming paper now to help maximize its impact. If they'll be preparing press materials, please inform our press team within the next 48 hours. Your manuscript will remain under strict press embargo until 2 pm Eastern Time on the date of publication. For more information, please contact onepress@plos.org.

You will receive an invoice from PLOS for your publication fee after your manuscript has reached the completed accept phase. If you receive an email requesting payment before acceptance or for any other service, this may be a phishing scheme. Learn how to identify phishing emails and protect your accounts at https://explore.plos.org/phishing.

If we can help with anything else, please email us at customercare@plos.org.

Thank you for submitting your work to PLOS ONE and supporting open access.

Kind regards,

PLOS ONE Editorial Office Staff

on behalf of

Dr. Gabriele Saretzki

Academic Editor

PLOS One
